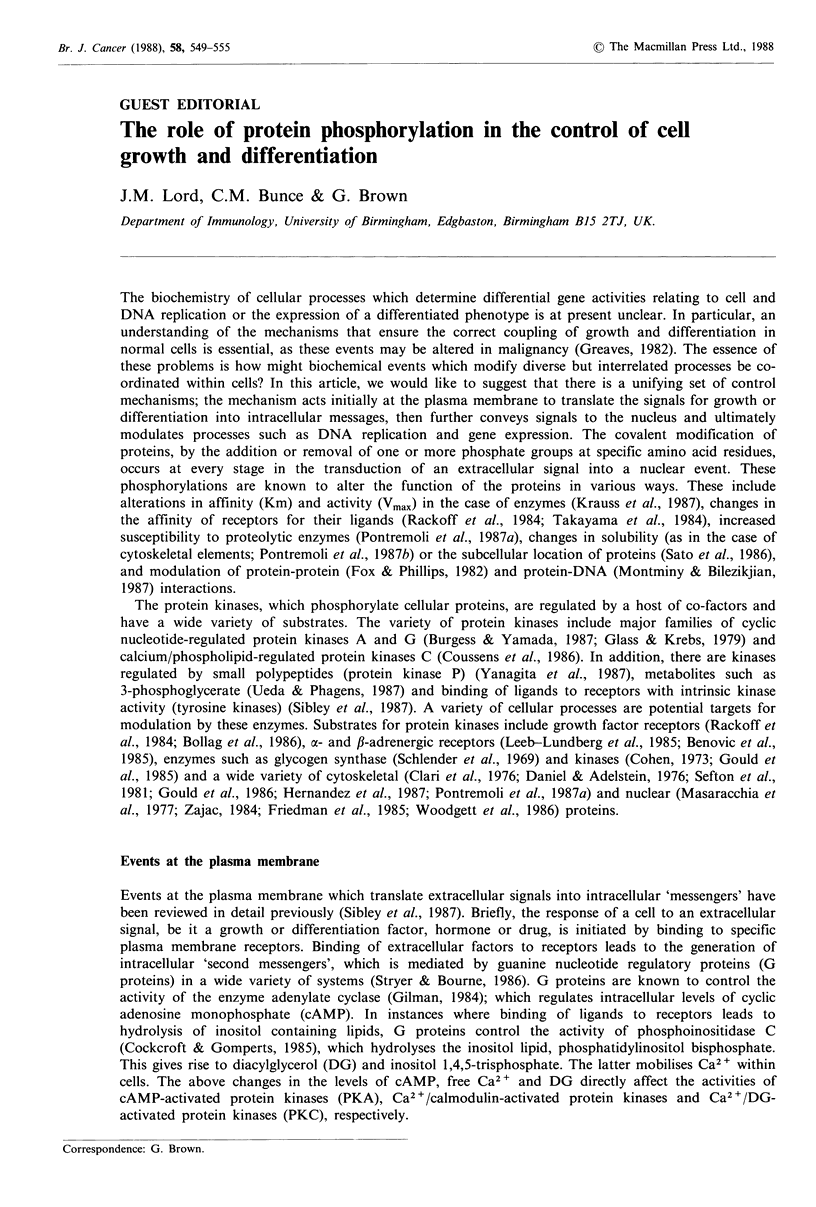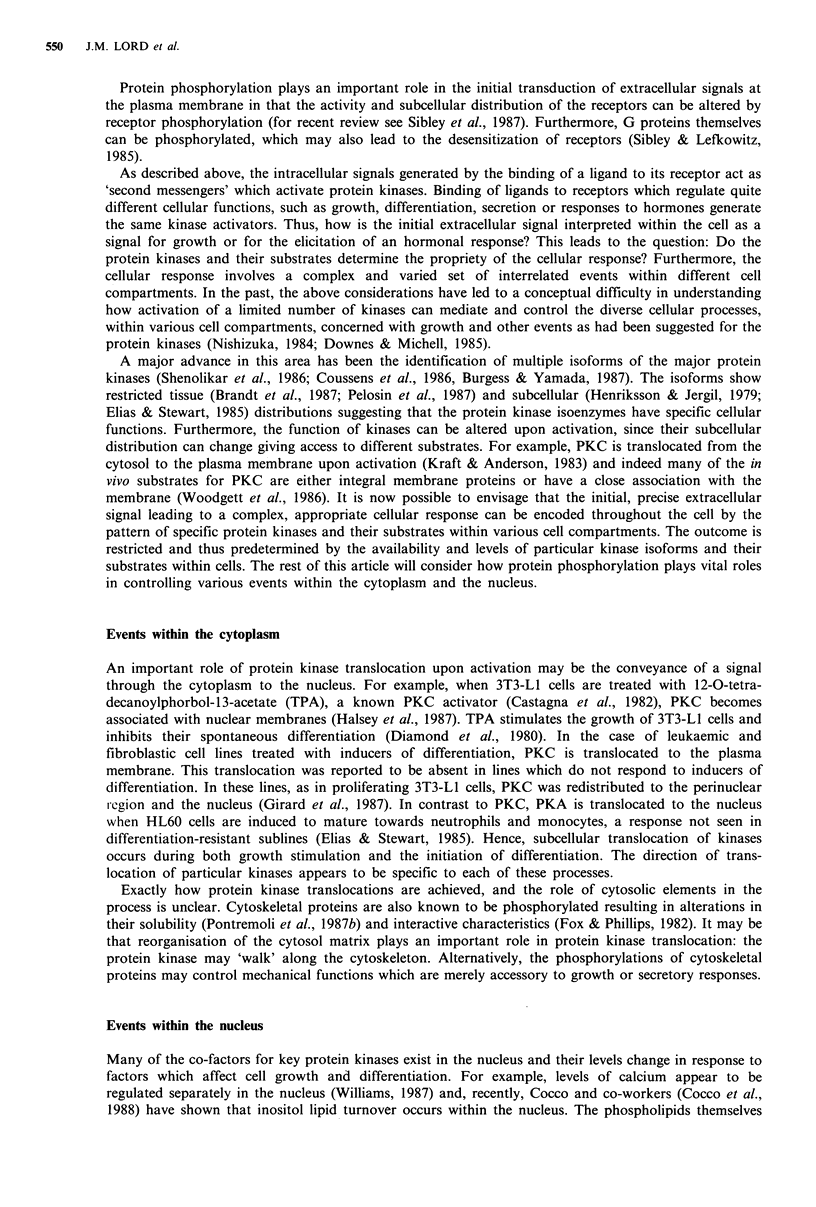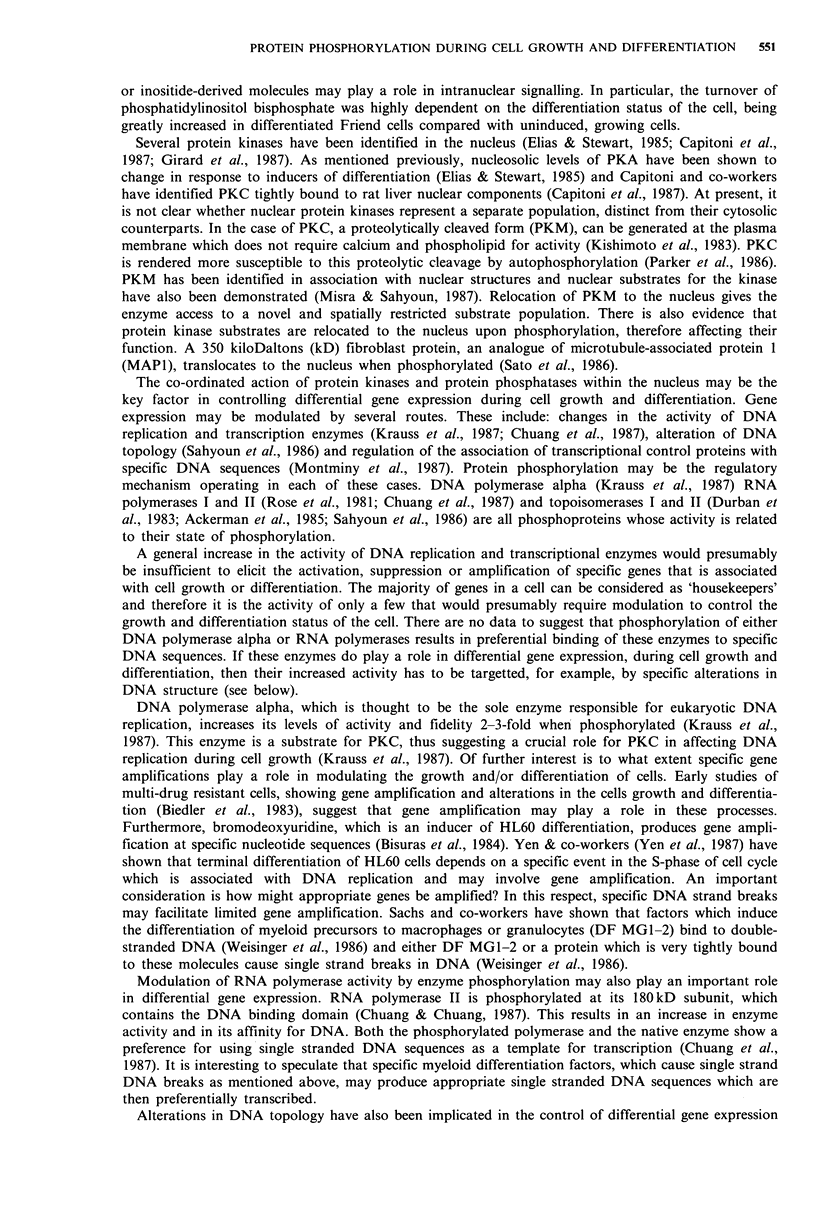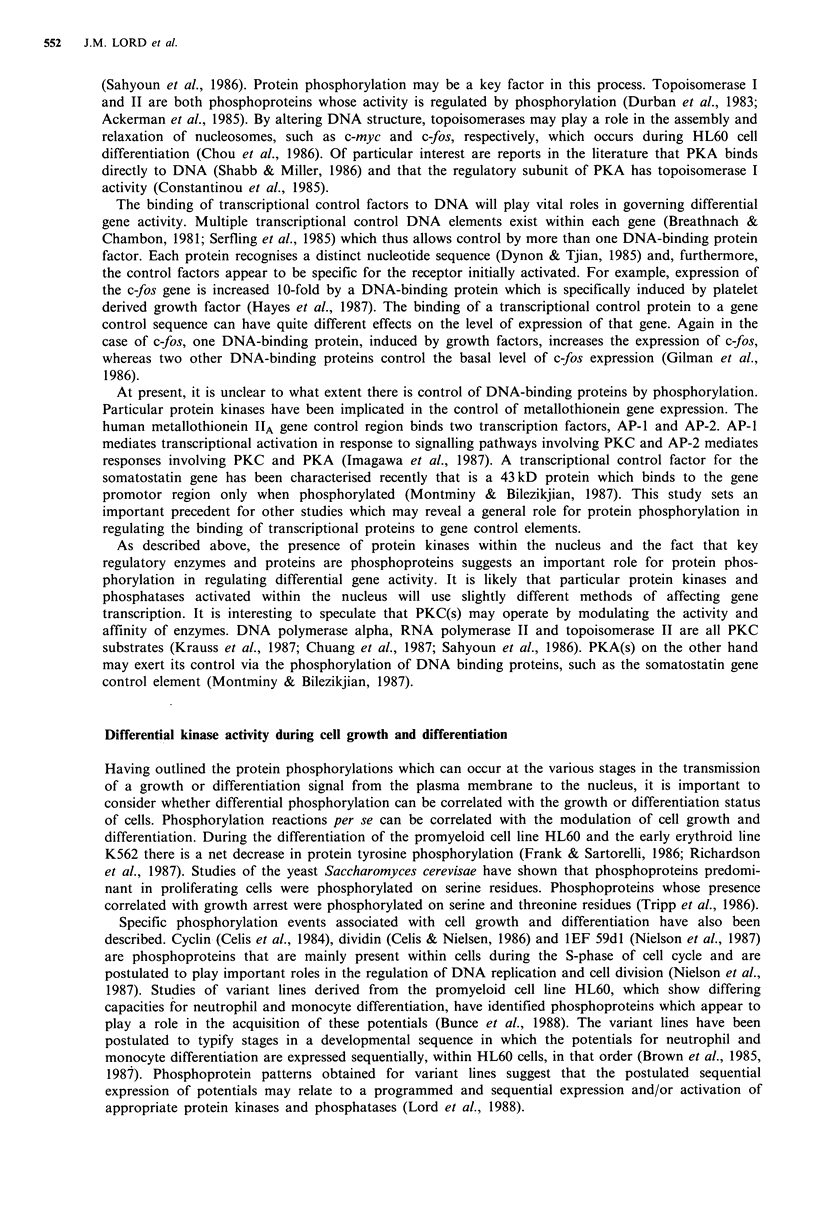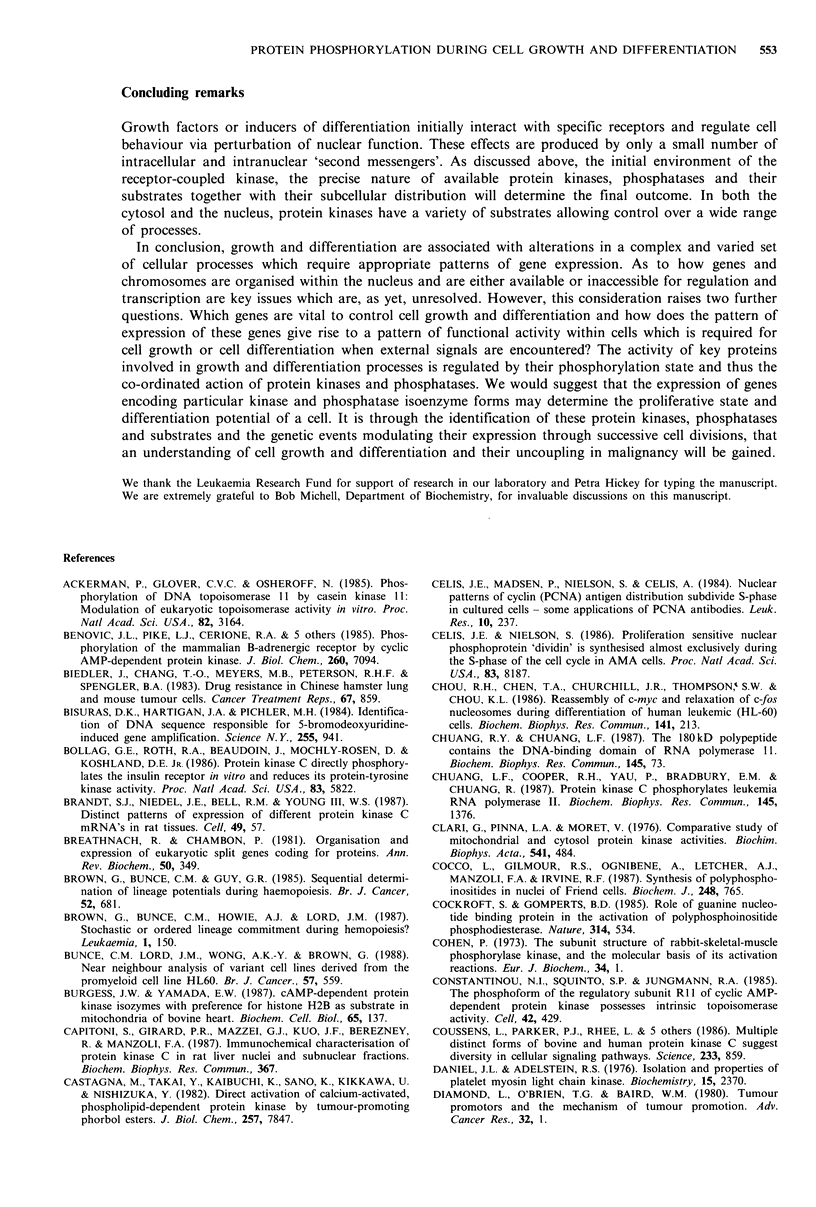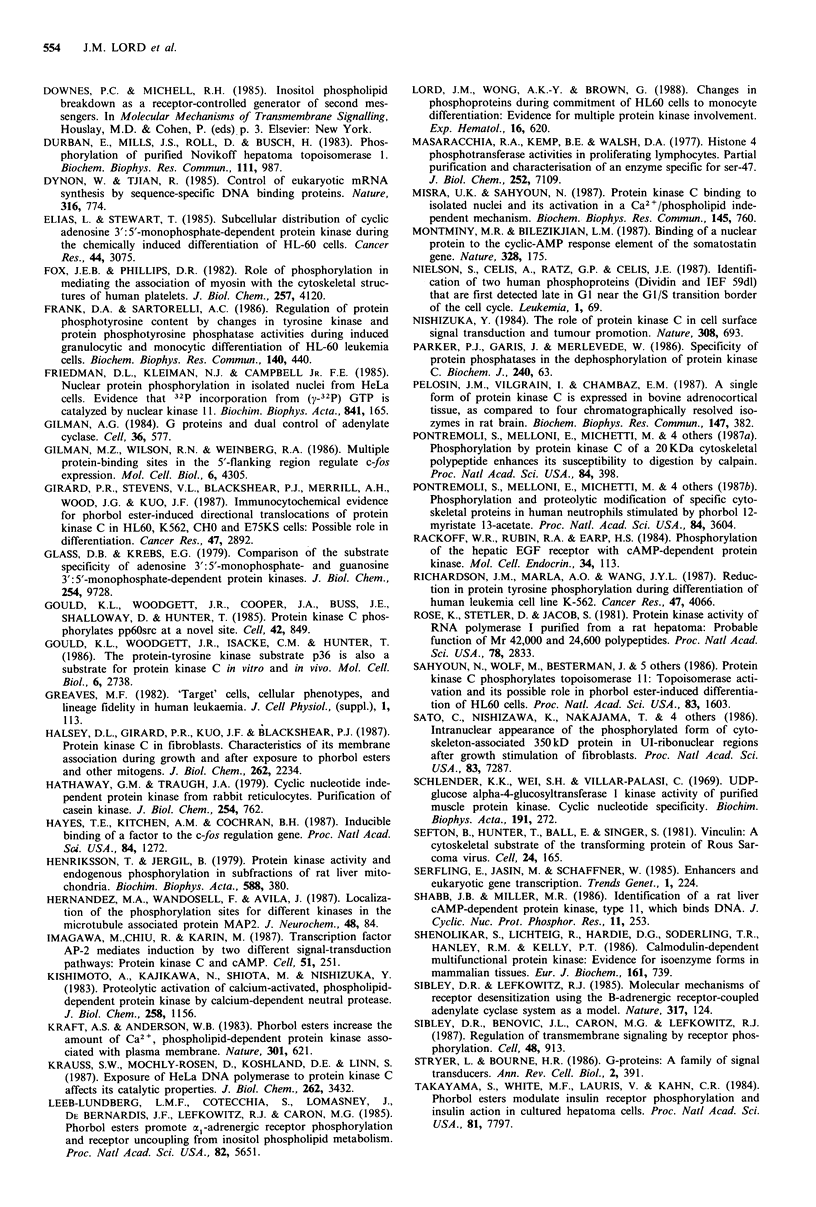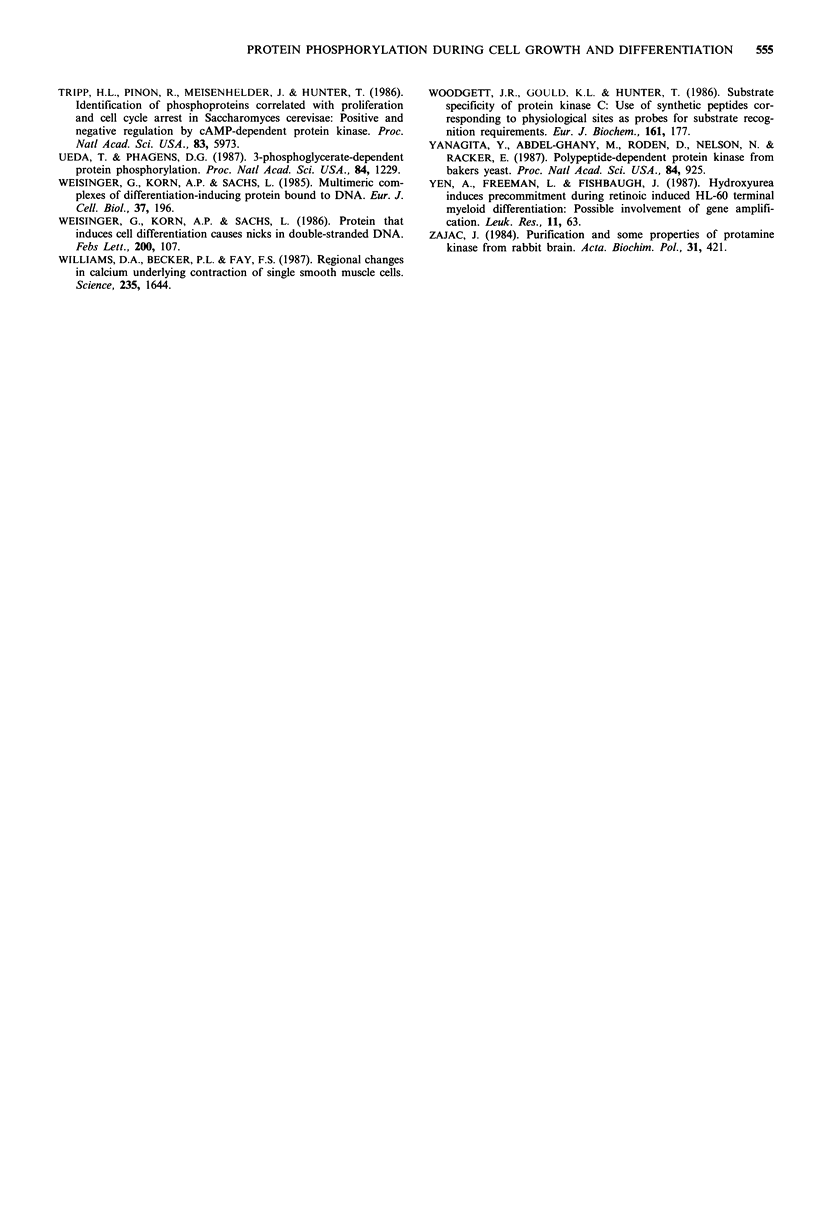# The role of protein phosphorylation in the control of cell growth and differentiation.

**DOI:** 10.1038/bjc.1988.256

**Published:** 1988-11

**Authors:** J. M. Lord, C. M. Bunce, G. Brown

**Affiliations:** Department of Immunology, University of Birmingham, Edgbaston, UK.


Br. J. Cancer (1988), 58, 549–555 © The Macmillan Press Ltd., 1988

GUEST EDITORIAL

### The role of protein phosphorylation in the control of cell growth and differentiation

J.M. Lord, C.M. Bunce & G. Brown

*Department of Immunology, University of Birmingham, Edgbaston, Birmingham B15 2TJ, UK.*

The biochemistry of cellular processes which determine differential gene activities relating to cell and DNA replication or the expression of a differentiated phenotype is at present unclear. In particular, an understanding of the mechanisms that ensure the correct coupling of growth and differentiation in normal cells is essential, as these events may be altered in malignancy (Greaves, 1982). The essence of these problems is how might biochemical events which modify diverse but interrelated processes be co-ordinated within cells? In this article, we would like to suggest that there is a unifying set of control mechanisms; the mechanism acts initially at the plasma membrane to translate the signals for growth or differentiation into intracellular messages, then further conveys signals to the nucleus and ultimately modulates processes such as DNA replication and gene expression. The covalent modification of proteins, by the addition or removal of one or more phosphate groups at specific amino acid residues, occurs at every stage in the transduction of an extracellular signal into a nuclear event. These phosphorylations are known to alter the function of the proteins in various ways. These include alterations in affinity (Km) and activity ($V_{max}$) in the case of enzymes (Krauss *et al.*, 1987), changes in the affinity of receptors for their ligands (Rackoff *et al.*, 1984; Takayama *et al.*, 1984), increased susceptibility to proteolytic enzymes (Pontremoli *et al.*, 1987*a*), changes in solubility (as in the case of cytoskeletal elements; Pontremoli *et al.*, 1987*b*) or the subcellular location of proteins (Sato *et al.*, 1986), and modulation of protein-protein (Fox & Phillips, 1982) and protein-DNA (Montminy & Bilezikjian, 1987) interactions.

The protein kinases, which phosphorylate cellular proteins, are regulated by a host of co-factors and have a wide variety of substrates. The variety of protein kinases include major families of cyclic nucleotide-regulated protein kinases A and G (Burgess & Yamada, 1987; Glass & Krebs, 1979) and calcium/phospholipid-regulated protein kinases C (Coussens *et al.*, 1986). In addition, there are kinases regulated by small polypeptides (protein kinase P) (Yanagita *et al.*, 1987), metabolites such as 3-phosphoglycerate (Ueda & Phagens, 1987) and binding of ligands to receptors with intrinsic kinase activity (tyrosine kinases) (Sibley *et al.*, 1987). A variety of cellular processes are potential targets for modulation by these enzymes. Substrates for protein kinases include growth factor receptors (Rackoff *et al.*, 1984; Bollag *et al.*, 1986), α- and β-adrenergic receptors (Leeb–Lundberg *et al.*, 1985; Benovic *et al.*, 1985), enzymes such as glycogen synthase (Schlender *et al.*, 1969) and kinases (Cohen, 1973; Gould *et al.*, 1985) and a wide variety of cytoskeletal (Clari *et al.*, 1976; Daniel & Adelstein, 1976; Sefton *et al.*, 1981; Gould *et al.*, 1986; Hernandez *et al.*, 1987; Pontremoli *et al.*, 1987*a*) and nuclear (Masaracchia *et al.*, 1977; Zajac, 1984; Friedman *et al.*, 1985; Woodgett *et al.*, 1986) proteins.

### Events at the plasma membrane

Events at the plasma membrane which translate extracellular signals into intracellular 'messengers' have been reviewed in detail previously (Sibley *et al.*, 1987). Briefly, the response of a cell to an extracellular signal, be it a growth or differentiation factor, hormone or drug, is initiated by binding to specific plasma membrane receptors. Binding of extracellular factors to receptors leads to the generation of intracellular 'second messengers', which is mediated by guanine nucleotide regulatory proteins (G proteins) in a wide variety of systems (Stryer & Bourne, 1986). G proteins are known to control the activity of the enzyme adenylate cyclase (Gilman, 1984); which regulates intracellular levels of cyclic adenosine monophosphate (cAMP). In instances where binding of ligands to receptors leads to hydrolysis of inositol containing lipids, G proteins control the activity of phosphoinositidase C (Cockcroft & Gomperts, 1985), which hydrolyses the inositol lipid, phosphatidylinositol bisphosphate. This gives rise to diacylglycerol (DG) and inositol 1,4,5-trisphosphate. The latter mobilises $Ca^{2+}$ within cells. The above changes in the levels of cAMP, free $Ca^{2+}$ and DG directly affect the activities of cAMP-activated protein kinases (PKA), $Ca^{2+}$/calmodulin-activated protein kinases and $Ca^{2+}$/DG-activated protein kinases (PKC), respectively.

Correspondence: G. Brown.

Protein phosphorylation plays an important role in the initial transduction of extracellular signals at the plasma membrane in that the activity and subcellular distribution of the receptors can be altered by receptor phosphorylation (for recent review see Sibley *et al.*, 1987). Furthermore, G proteins themselves can be phosphorylated, which may also lead to the desensitization of receptors (Sibley & Lefkowitz, 1985).

As described above, the intracellular signals generated by the binding of a ligand to its receptor act as 'second messengers' which activate protein kinases. Binding of ligands to receptors which regulate quite different cellular functions, such as growth, differentiation, secretion or responses to hormones generate the same kinase activators. Thus, how is the initial extracellular signal interpreted within the cell as a signal for growth or for the elicitation of an hormonal response? This leads to the question: Do the protein kinases and their substrates determine the propriety of the cellular response? Furthermore, the cellular response involves a complex and varied set of interrelated events within different cell compartments. In the past, the above considerations have led to a conceptual difficulty in understanding how activation of a limited number of kinases can mediate and control the diverse cellular processes, within various cell compartments, concerned with growth and other events as had been suggested for the protein kinases (Nishizuka, 1984; Downes & Michell, 1985).

A major advance in this area has been the identification of multiple isoforms of the major protein kinases (Shenolikar *et al.*, 1986; Coussens *et al.*, 1986, Burgess & Yamada, 1987). The isoforms show restricted tissue (Brandt *et al.*, 1987; Pelosin *et al.*, 1987) and subcellular (Henriksson & Jergil, 1979; Elias & Stewart, 1985) distributions suggesting that the protein kinase isoenzymes have specific cellular functions. Furthermore, the function of kinases can be altered upon activation, since their subcellular distribution can change giving access to different substrates. For example, PKC is translocated from the cytosol to the plasma membrane upon activation (Kraft & Anderson, 1983) and indeed many of the *in vivo* substrates for PKC are either integral membrane proteins or have a close association with the membrane (Woodgett *et al.*, 1986). It is now possible to envisage that the initial, precise extracellular signal leading to a complex, appropriate cellular response can be encoded throughout the cell by the pattern of specific protein kinases and their substrates within various cell compartments. The outcome is restricted and thus predetermined by the availability and levels of particular kinase isoforms and their substrates within cells. The rest of this article will consider how protein phosphorylation plays vital roles in controlling various events within the cytoplasm and the nucleus.

### Events within the cytoplasm

An important role of protein kinase translocation upon activation may be the conveyance of a signal through the cytoplasm to the nucleus. For example, when 3T3-L1 cells are treated with 12-O-tetradecanoylphorbol-13-acetate (TPA), a known PKC activator (Castagna *et al.*, 1982), PKC becomes associated with nuclear membranes (Halsey *et al.*, 1987). TPA stimulates the growth of 3T3-L1 cells and inhibits their spontaneous differentiation (Diamond *et al.*, 1980). In the case of leukaemic and fibroblastic cell lines treated with inducers of differentiation, PKC is translocated to the plasma membrane. This translocation was reported to be absent in lines which do not respond to inducers of differentiation. In these lines, as in proliferating 3T3-L1 cells, PKC was redistributed to the perinuclear region and the nucleus (Girard *et al.*, 1987). In contrast to PKC, PKA is translocated to the nucleus when HL60 cells are induced to mature towards neutrophils and monocytes, a response not seen in differentiation-resistant sublines (Elias & Stewart, 1985). Hence, subcellular translocation of kinases occurs during both growth stimulation and the initiation of differentiation. The direction of translocation of particular kinases appears to be specific to each of these processes.

Exactly how protein kinase translocations are achieved, and the role of cytosolic elements in the process is unclear. Cytoskeletal proteins are also known to be phosphorylated resulting in alterations in their solubility (Pontremoli *et al.*, 1987*b*) and interactive characteristics (Fox & Phillips, 1982). It may be that reorganisation of the cytosol matrix plays an important role in protein kinase translocation: the protein kinase may 'walk' along the cytoskeleton. Alternatively, the phosphorylations of cytoskeletal proteins may control mechanical functions which are merely accessory to growth or secretory responses.

### Events within the nucleus

Many of the co-factors for key protein kinases exist in the nucleus and their levels change in response to factors which affect cell growth and differentiation. For example, levels of calcium appear to be regulated separately in the nucleus (Williams, 1987) and, recently, Cocco and co-workers (Cocco *et al.*, 1988) have shown that inositol lipid turnover occurs within the nucleus. The phospholipids themselves or inositide-derived molecules may play a role in intranuclear signalling. In particular, the turnover of phosphatidylinositol bisphosphate was highly dependent on the differentiation status of the cell, being greatly increased in differentiated Friend cells compared with uninduced, growing cells.

Several protein kinases have been identified in the nucleus (Elias & Stewart, 1985; Capitoni *et al.*, 1987; Girard *et al.*, 1987). As mentioned previously, nucleosolic levels of PKA have been shown to change in response to inducers of differentiation (Elias & Stewart, 1985) and Capitoni and co-workers have identified PKC tightly bound to rat liver nuclear components (Capitoni *et al.*, 1987). At present, it is not clear whether nuclear protein kinases represent a separate population, distinct from their cytosolic counterparts. In the case of PKC, a proteolytically cleaved form (PKM), can be generated at the plasma membrane which does not require calcium and phospholipid for activity (Kishimoto *et al.*, 1983). PKC is rendered more susceptible to this proteolytic cleavage by autophosphorylation (Parker *et al.*, 1986). PKM has been identified in association with nuclear structures and nuclear substrates for the kinase have also been demonstrated (Misra & Sahyoun, 1987). Relocation of PKM to the nucleus gives the enzyme access to a novel and spatially restricted substrate population. There is also evidence that protein kinase substrates are relocated to the nucleus upon phosphorylation, therefore affecting their function. A 350 kiloDaltons (kD) fibroblast protein, an analogue of microtubule-associated protein 1 (MAP1), translocates to the nucleus when phosphorylated (Sato *et al.*, 1986).

The co-ordinated action of protein kinases and protein phosphatases within the nucleus may be the key factor in controlling differential gene expression during cell growth and differentiation. Gene expression may be modulated by several routes. These include: changes in the activity of DNA replication and transcription enzymes (Krauss *et al.*, 1987; Chuang *et al.*, 1987), alteration of DNA topology (Sahyoun *et al.*, 1986) and regulation of the association of transcriptional control proteins with specific DNA sequences (Montminy *et al.*, 1987). Protein phosphorylation may be the regulatory mechanism operating in each of these cases. DNA polymerase alpha (Krauss *et al.*, 1987) RNA polymerases I and II (Rose *et al.*, 1981; Chuang *et al.*, 1987) and topoisomerases I and II (Durban *et al.*, 1983; Ackerman *et al.*, 1985; Sahyoun *et al.*, 1986) are all phosphoproteins whose activity is related to their state of phosphorylation.

A general increase in the activity of DNA replication and transcriptional enzymes would presumably be insufficient to elicit the activation, suppression or amplification of specific genes that is associated with cell growth or differentiation. The majority of genes in a cell can be considered as 'housekeepers' and therefore it is the activity of only a few that would presumably require modulation to control the growth and differentiation status of the cell. There are no data to suggest that phosphorylation of either DNA polymerase alpha or RNA polymerases results in preferential binding of these enzymes to specific DNA sequences. If these enzymes do play a role in differential gene expression, during cell growth and differentiation, then their increased activity has to be targetted, for example, by specific alterations in DNA structure (see below).

DNA polymerase alpha, which is thought to be the sole enzyme responsible for eukaryotic DNA replication, increases its levels of activity and fidelity 2–3-fold when phosphorylated (Krauss *et al.*, 1987). This enzyme is a substrate for PKC, thus suggesting a crucial role for PKC in affecting DNA replication during cell growth (Krauss *et al.*, 1987). Of further interest is to what extent specific gene amplifications play a role in modulating the growth and/or differentiation of cells. Early studies of multi-drug resistant cells, showing gene amplification and alterations in the cells growth and differentiation (Biedler *et al.*, 1983), suggest that gene amplification may play a role in these processes. Furthermore, bromodeoxyuridine, which is an inducer of HL60 differentiation, produces gene amplification at specific nucleotide sequences (Bisuras *et al.*, 1984). Yen & co-workers (Yen *et al.*, 1987) have shown that terminal differentiation of HL60 cells depends on a specific event in the S-phase of cell cycle which is associated with DNA replication and may involve gene amplification. An important consideration is how might appropriate genes be amplified? In this respect, specific DNA strand breaks may facilitate limited gene amplification. Sachs and co-workers have shown that factors which induce the differentiation of myeloid precursors to macrophages or granulocytes (DF MG1–2) bind to double-stranded DNA (Weisinger *et al.*, 1986) and either DF MG1–2 or a protein which is very tightly bound to these molecules cause single strand breaks in DNA (Weisinger *et al.*, 1986).

Modulation of RNA polymerase activity by enzyme phosphorylation may also play an important role in differential gene expression. RNA polymerase II is phosphorylated at its 180 kD subunit, which contains the DNA binding domain (Chuang & Chuang, 1987). This results in an increase in enzyme activity and in its affinity for DNA. Both the phosphorylated polymerase and the native enzyme show a preference for using single stranded DNA sequences as a template for transcription (Chuang *et al.*, 1987). It is interesting to speculate that specific myeloid differentiation factors, which cause single strand DNA breaks as mentioned above, may produce appropriate single stranded DNA sequences which are then preferentially transcribed.

Alterations in DNA topology have also been implicated in the control of differential gene expression

(Sahyoun *et al.*, 1986). Protein phosphorylation may be a key factor in this process. Topoisomerase I and II are both phosphoproteins whose activity is regulated by phosphorylation (Durban *et al.*, 1983; Ackerman *et al.*, 1985). By altering DNA structure, topoisomerases may play a role in the assembly and relaxation of nucleosomes, such as c-*myc* and c-*fos*, respectively, which occurs during HL60 cell differentiation (Chou *et al.*, 1986). Of particular interest are reports in the literature that PKA binds directly to DNA (Shabb & Miller, 1986) and that the regulatory subunit of PKA has topoisomerase I activity (Constantinou *et al.*, 1985).

The binding of transcriptional control factors to DNA will play vital roles in governing differential gene activity. Multiple transcriptional control DNA elements exist within each gene (Breathnach & Chambon, 1981; Serfling *et al.*, 1985) which thus allows control by more than one DNA-binding protein factor. Each protein recognises a distinct nucleotide sequence (Dynon & Tjian, 1985) and, furthermore, the control factors appear to be specific for the receptor initially activated. For example, expression of the c-*fos* gene is increased 10-fold by a DNA-binding protein which is specifically induced by platelet derived growth factor (Hayes *et al.*, 1987). The binding of a transcriptional control protein to a gene control sequence can have quite different effects on the level of expression of that gene. Again in the case of c-*fos*, one DNA-binding protein, induced by growth factors, increases the expression of c-*fos*, whereas two other DNA-binding proteins control the basal level of c-*fos* expression (Gilman *et al.*, 1986).

At present, it is unclear to what extent there is control of DNA-binding proteins by phosphorylation. Particular protein kinases have been implicated in the control of metallothionein gene expression. The human metallothionein $II_A$ gene control region binds two transcription factors, AP-1 and AP-2. AP-1 mediates transcriptional activation in response to signalling pathways involving PKC and AP-2 mediates responses involving PKC and PKA (Imagawa *et al.*, 1987). A transcriptional control factor for the somatostatin gene has been characterised recently that is a 43 kD protein which binds to the gene promotor region only when phosphorylated (Montminy & Bilezikjian, 1987). This study sets an important precedent for other studies which may reveal a general role for protein phosphorylation in regulating the binding of transcriptional proteins to gene control elements.

As described above, the presence of protein kinases within the nucleus and the fact that key regulatory enzymes and proteins are phosphoproteins suggests an important role for protein phosphorylation in regulating differential gene activity. It is likely that particular protein kinases and phosphatases activated within the nucleus will use slightly different methods of affecting gene transcription. It is interesting to speculate that PKC(s) may operate by modulating the activity and affinity of enzymes. DNA polymerase alpha, RNA polymerase II and topoisomerase II are all PKC substrates (Krauss *et al.*, 1987; Chuang *et al.*, 1987; Sahyoun *et al.*, 1986). PKA(s) on the other hand may exert its control via the phosphorylation of DNA binding proteins, such as the somatostatin gene control element (Montminy & Bilezikjian, 1987).

### Differential kinase activity during cell growth and differentiation

Having outlined the protein phosphorylations which can occur at the various stages in the transmission of a growth or differentiation signal from the plasma membrane to the nucleus, it is important to consider whether differential phosphorylation can be correlated with the growth or differentiation status of cells. Phosphorylation reactions *per se* can be correlated with the modulation of cell growth and differentiation. During the differentiation of the promyeloid cell line HL60 and the early erythroid line K562 there is a net decrease in protein tyrosine phosphorylation (Frank & Sartorelli, 1986; Richardson *et al.*, 1987). Studies of the yeast *Saccharomyces cerevisae* have shown that phosphoproteins predominant in proliferating cells were phosphorylated on serine residues. Phosphoproteins whose presence correlated with growth arrest were phosphorylated on serine and threonine residues (Tripp *et al.*, 1986).

Specific phosphorylation events associated with cell growth and differentiation have also been described. Cyclin (Celis *et al.*, 1984), dividin (Celis & Nielsen, 1986) and 1EF 59d1 (Nielson *et al.*, 1987) are phosphoproteins that are mainly present within cells during the S-phase of cell cycle and are postulated to play important roles in the regulation of DNA replication and cell division (Nielson *et al.*, 1987). Studies of variant lines derived from the promyeloid cell line HL60, which show differing capacities for neutrophil and monocyte differentiation, have identified phosphoproteins which appear to play a role in the acquisition of these potentials (Bunce *et al.*, 1988). The variant lines have been postulated to typify stages in a developmental sequence in which the potentials for neutrophil and monocyte differentiation are expressed sequentially, within HL60 cells, in that order (Brown *et al.*, 1985, 1987). Phosphoprotein patterns obtained for variant lines suggest that the postulated sequential expression of potentials may relate to a programmed and sequential expression and/or activation of appropriate protein kinases and phosphatases (Lord *et al.*, 1988).

### Concluding remarks

Growth factors or inducers of differentiation initially interact with specific receptors and regulate cell behaviour via perturbation of nuclear function. These effects are produced by only a small number of intracellular and intranuclear 'second messengers'. As discussed above, the initial environment of the receptor-coupled kinase, the precise nature of available protein kinases, phosphatases and their substrates together with their subcellular distribution will determine the final outcome. In both the cytosol and the nucleus, protein kinases have a variety of substrates allowing control over a wide range of processes.

In conclusion, growth and differentiation are associated with alterations in a complex and varied set of cellular processes which require appropriate patterns of gene expression. As to how genes and chromosomes are organised within the nucleus and are either available or inaccessible for regulation and transcription are key issues which are, as yet, unresolved. However, this consideration raises two further questions. Which genes are vital to control cell growth and differentiation and how does the pattern of expression of these genes give rise to a pattern of functional activity within cells which is required for cell growth or cell differentiation when external signals are encountered? The activity of key proteins involved in growth and differentiation processes is regulated by their phosphorylation state and thus the co-ordinated action of protein kinases and phosphatases. We would suggest that the expression of genes encoding particular kinase and phosphatase isoenzyme forms may determine the proliferative state and differentiation potential of a cell. It is through the identification of these protein kinases, phosphatases and substrates and the genetic events modulating their expression through successive cell divisions, that an understanding of cell growth and differentiation and their uncoupling in malignancy will be gained.

We thank the Leukaemia Research Fund for support of research in our laboratory and Petra Hickey for typing the manuscript. We are extremely grateful to Bob Michell, Department of Biochemistry, for invaluable discussions on this manuscript.

### References

ACKERMAN, P., GLOVER, C.V.C. & OSHEROFF, N. (1985). Phosphorylation of DNA topoisomerase 11 by casein kinase 11: Modulation of eukaryotic topoisomerase activity *in vitro*. *Proc. Natl Acad. Sci. USA.*, **82**, 3164.

BENOVIC, J.L., PIKE, L.J., CERIONE, R.A. & 5 others (1985). Phosphorylation of the mammalian B-adrenergic receptor by cyclic AMP-dependent protein kinase. *J. Biol. Chem.*, **260**, 7094.

BIEDLER, J., CHANG, T.-O., MEYERS, M.B., PETERSON, R.H.F. & SPENGLER, B.A. (1983). Drug resistance in Chinese hamster lung and mouse tumour cells. *Cancer Treatment Reps.*, **67**, 859.

BISURAS, D.K., HARTIGAN, J.A. & PICHLER, M.H. (1984). Identification of DNA sequence responsible for 5-bromodeoxyuridine-induced gene amplification. *Science N.Y.*, **255**, 941.

BOLLAG, G.E., ROTH, R.A., BEAUDOIN, J., MOCHLY-ROSEN, D. & KOSHLAND, D.E. JR. (1986). Protein kinase C directly phosphorylates the insulin receptor *in vitro* and reduces its protein-tyrosine kinase activity. *Proc. Natl Acad. Sci. USA.*, **83**, 5822.

BRANDT, S.J., NIEDEL, J.E., BELL, R.M. & YOUNG III, W.S. (1987). Distinct patterns of expression of different protein kinase C mRNA's in rat tissues. *Cell*, **49**, 57.

BREATHNACH, R. & CHAMBON, P. (1981). Organisation and expression of eukaryotic split genes coding for proteins. *Ann. Rev. Biochem.*, **50**, 349.

BROWN, G., BUNCE, C.M. & GUY, G.R. (1985). Sequential determination of lineage potentials during haemopoiesis. *Br. J. Cancer*, **52**, 681.

BROWN, G., BUNCE, C.M., HOWIE, A.J. & LORD, J.M. (1987). Stochastic or ordered lineage commitment during hemopoiesis? *Leukaemia*, **1**, 150.

BUNCE, C.M. LORD, J.M., WONG, A.K.-Y. & BROWN, G. (1988). Near neighbour analysis of variant cell lines derived from the promyeloid cell line HL60. *Br. J. Cancer.*, **57**, 559.

BURGESS, J.W. & YAMADA, E.W. (1987). cAMP-dependent protein kinase isozymes with preference for histone H2B as substrate in mitochondria of bovine heart. *Biochem. Cell. Biol.*, **65**, 137.

CAPITONI, S., GIRARD, P.R., MAZZEI, G.J., KUO, J.F., BEREZNEY, R. & MANZOLI, F.A. (1987). Immunochemical characterisation of protein kinase C in rat liver nuclei and subnuclear fractions. *Biochem. Biophys. Res. Commun.*, **367**.

CASTAGNA, M., TAKAI, Y., KAIBUCHI, K., SANO, K., KIKKAWA, U. & NISHIZUKA, Y. (1982). Direct activation of calcium-activated, phospholipid-dependent protein kinase by tumour-promoting phorbol esters. *J. Biol. Chem.*, **257**, 7847.

CELIS, J.E., MADSEN, P., NIELSON, S. & CELIS, A. (1984). Nuclear patterns of cyclin (PCNA) antigen distribution subdivide S-phase in cultured cells – some applications of PCNA antibodies. *Leuk. Res.*, **10**, 237.

CELIS, J.E. & NIELSON, S. (1986). Proliferation sensitive nuclear phosphoprotein 'dividin' is synthesised almost exclusively during the S-phase of the cell cycle in AMA cells. *Proc. Natl Acad. Sci. USA.*, **83**, 8187.

CHOU, R.H., CHEN, T.A., CHURCHILL, J.R., THOMPSON, S.W. & CHOU, K.L. (1986). Reassembly of c-*myc* and relaxation of c-*fos* nucleosomes during differentiation of human leukemic (HL-60) cells. *Biochem. Biophys. Res. Commun.*, **141**, 213.

CHUANG, R.Y. & CHUANG, L.F. (1987). The 180 kD polypeptide contains the DNA-binding domain of RNA polymerase 11. *Biochem. Biophys. Res. Commun.*, **145**, 73.

CHUANG, L.F., COOPER, R.H., YAU, P., BRADBURY, E.M. & CHUANG, R. (1987). Protein kinase C phosphorylates leukemia RNA polymerase II. *Biochem. Biophys. Res. Commun.*, **145**, 1376.

CLARI, G., PINNA, L.A. & MORET, V. (1976). Comparative study of mitochondrial and cytosol protein kinase activities. *Biochim. Biophys. Acta.*, **541**, 484.

COCCO, L., GILMOUR, R.S., OGNIBENE, A., LETCHER, A.J., MANZOLI, F.A. & IRVINE, R.F. (1987). Synthesis of polyphosphoinositides in nuclei of Friend cells. *Biochem. J.*, **248**, 765.

COCKROFT, S. & GOMPERTS, B.D. (1985). Role of guanine nucleotide binding protein in the activation of polyphosphoinositide phosphodiesterase. *Nature*, **314**, 534.

COHEN, P. (1973). The subunit structure of rabbit-skeletal-muscle phosphorylase kinase, and the molecular basis of its activation reactions. *Eur. J. Biochem.*, **34**, 1.

CONSTANTINOU, N.I., SQUINTO, S.P. & JUNGMANN, R.A. (1985). The phosphoform of the regulatory subunit R11 of cyclic AMP-dependent protein kinase possesses intrinsic topoisomerase activity. *Cell*, **42**, 429.

COUSSENS, L., PARKER, P.J., RHEE, L. & 5 others (1986). Multiple distinct forms of bovine and human protein kinase C suggest diversity in cellular signaling pathways. *Science*, **233**, 859.

DANIEL, J.L. & ADELSTEIN, R.S. (1976). Isolation and properties of platelet myosin light chain kinase. *Biochemistry*, **15**, 2370.

DIAMOND, L., O'BRIEN, T.G. & BAIRD, W.M. (1980). Tumour promotors and the mechanism of tumour promotion. *Adv. Cancer Res.*, **32**, 1.

DOWNES, P.C. & MICHELL, R.H. (1985). Inositol phospholipid breakdown as a receptor-controlled generator of second messengers. In *Molecular Mechanisms of Transmembrane Signalling*, Houslay, M.D. & Cohen, P. (eds) p. 3. Elsevier: New York.

DURBAN, E., MILLS, J.S., ROLL, D. & BUSCH, H. (1983). Phosphorylation of purified Novikoff hepatoma topoisomerase 1. *Biochem. Biophys. Res. Commun.*, **111**, 987.

DYNON, W. & TJIAN, R. (1985). Control of eukaryotic mRNA synthesis by sequence-specific DNA binding proteins. *Nature*, **316**, 774.

ELIAS, L. & STEWART, T. (1985). Subcellular distribution of cyclic adenosine 3′:5′-monophosphate-dependent protein kinase during the chemically induced differentiation of HL-60 cells. *Cancer Res.*, **44**, 3075.

FOX, J.E.B. & PHILLIPS, D.R. (1982). Role of phosphorylation in mediating the association of myosin with the cytoskeletal structures of human platelets. *J. Biol. Chem.*, **257**, 4120.

FRANK, D.A. & SARTORELLI, A.C. (1986). Regulation of protein phosphotyrosine content by changes in tyrosine kinase and protein phosphotyrosine phosphatase activities during induced granulocytic and monocytic differentiation of HL-60 leukemia cells. *Biochem. Biophys. Res. Commun.*, **140**, 440.

FRIEDMAN, D.L., KLEIMAN, N.J. & CAMPBELL JR. F.E. (1985). Nuclear protein phosphorylation in isolated nuclei from HeLa cells. Evidence that $^{32}P$ incorporation from ($\gamma$-$^{32}P$) GTP is catalyzed by nuclear kinase 11. *Biochim. Biophys. Acta.*, **841**, 165.

GILMAN, A.G. (1984). G proteins and dual control of adenylate cyclase. *Cell*, **36**, 577.

GILMAN, M.Z., WILSON, R.N. & WEINBERG, R.A. (1986). Multiple protein-binding sites in the 5′-flanking region regulate c-*fos* expression. *Mol. Cell. Biol.*, **6**, 4305.

GIRARD, P.R., STEVENS, V.L., BLACKSHEAR, P.J., MERRILL, A.H., WOOD, J.G. & KUO, J.F. (1987). Immunocytochemical evidence for phorbol ester-induced directional translocations of protein kinase C in HL60, K562, CH0 and E75KS cells: Possible role in differentiation. *Cancer Res.*, **47**, 2892.

GLASS, D.B. & KREBS, E.G. (1979). Comparison of the substrate specificity of adenosine 3′:5′-monophosphate- and guanosine 3′:5′-monophosphate-dependent protein kinases. *J. Biol. Chem.*, **254**, 9728.

GOULD, K.L., WOODGETT, J.R., COOPER, J.A., BUSS, J.E., SHALLOWAY, D. & HUNTER, T. (1985). Protein kinase C phosphorylates pp60src at a novel site. *Cell*, **42**, 849.

GOULD, K.L., WOODGETT, J.R., ISACKE, C.M. & HUNTER, T. (1986). The protein-tyrosine kinase substrate p36 is also a substrate for protein kinase C *in vitro* and *in vivo*. *Mol. Cell. Biol.*, **6**, 2738.

GREAVES, M.F. (1982). 'Target' cells, cellular phenotypes, and lineage fidelity in human leukaemia. *J. Cell Physiol.*, (suppl.), **1**, 113.

HALSEY, D.L., GIRARD, P.R., KUO, J.F. & BLACKSHEAR, P.J. (1987). Protein kinase C in fibroblasts. Characteristics of its membrane association during growth and after exposure to phorbol esters and other mitogens. *J. Biol. Chem.*, **262**, 2234.

HATHAWAY, G.M. & TRAUGH, J.A. (1979). Cyclic nucleotide independent protein kinase from rabbit reticulocytes. Purification of casein kinase. *J. Biol. Chem.*, **254**, 762.

HAYES, T.E., KITCHEN, A.M. & COCHRAN, B.H. (1987). Inducible binding of a factor to the c-*fos* regulation gene. *Proc. Natl Acad. Sci. USA.*, **84**, 1272.

HENRIKSSON, T. & JERGIL, B. (1979). Protein kinase activity and endogenous phosphorylation in subfractions of rat liver mitochondria. *Biochim. Biophys. Acta.*, **588**, 380.

HERNANDEZ, M.A., WANDOSELL, F. & AVILA, J. (1987). Localization of the phosphorylation sites for different kinases in the microtubule associated protein MAP2. *J. Neurochem.*, **48**, 84.

IMAGAWA, M., CHIU, R. & KARIN, M. (1987). Transcription factor AP-2 mediates induction by two different signal-transduction pathways: Protein kinase C and cAMP. *Cell*, **51**, 251.

KISHIMOTO, A., KAJIKAWA, N., SHIOTA, M. & NISHIZUKA, Y. (1983). Proteolytic activation of calcium-activated, phospholipid-dependent protein kinase by calcium-dependent neutral protease. *J. Biol. Chem.*, **258**, 1156.

KRAFT, A.S. & ANDERSON, W.B. (1983). Phorbol esters increase the amount of $Ca^{2+}$, phospholipid-dependent protein kinase associated with plasma membrane. *Nature*, **301**, 621.

KRAUSS, S.W., MOCHLY-ROSEN, D., KOSHLAND, D.E. & LINN, S. (1987). Exposure of HeLa DNA polymerase to protein kinase C affects its catalytic properties. *J. Biol. Chem.*, **262**, 3432.

LEEB-LUNDBERG, L.M.F., COTECCHIA, S., LOMASNEY, J., DE BERNARDIS, J.F., LEFKOWITZ, R.J. & CARON, M.G. (1985). Phorbol esters promote $\alpha_1$-adrenergic receptor phosphorylation and receptor uncoupling from inositol phospholipid metabolism. *Proc. Natl Acad. Sci. USA.*, **82**, 5651.

LORD, J.M., WONG, A.K.-Y. & BROWN, G. (1988). Changes in phosphoproteins during commitment of HL60 cells to monocyte differentiation: Evidence for multiple protein kinase involvement. *Exp. Hematol.*, **16**, 620.

MASARACCHIA, R.A., KEMP, B.E. & WALSH, D.A. (1977). Histone 4 phosphotransferase activities in proliferating lymphocytes. Partial purification and characterisation of an enzyme specific for ser-47. *J. Biol. Chem.*, **252**, 7109.

MISRA, U.K. & SAHYOUN, N. (1987). Protein kinase C binding to isolated nuclei and its activation in a $Ca^{2+}$/phospholipid independent mechanism. *Biochem. Biophys. Res. Commun.*, **145**, 760.

MONTMINY, M.R. & BILEZIKJIAN, L.M. (1987). Binding of a nuclear protein to the cyclic-AMP response element of the somatostatin gene. *Nature*, **328**, 175.

NIELSON, S., CELIS, A., RATZ, G.P. & CELIS, J.E. (1987). Identification of two human phosphoproteins (Dividin and IEF 59dl) that are first detected late in G1 near the G1/S transition border of the cell cycle. *Leukemia*, **1**, 69.

NISHIZUKA, Y. (1984). The role of protein kinase C in cell surface signal transduction and tumour promotion. *Nature*, **308**, 693.

PARKER, P.J., GARIS, J. & MERLEVEDE, W. (1986). Specificity of protein phosphatases in the dephosphorylation of protein kinase C. *Biochem. J.*, **240**, 63.

PELOSIN, J.M., VILGRAIN, I. & CHAMBAZ, E.M. (1987). A single form of protein kinase C is expressed in bovine adrenocortical tissue, as compared to four chromatographically resolved isozymes in rat brain. *Biochem. Biophys. Res. Commun.*, **147**, 382.

PONTREMOLI, S., MELLONI, E., MICHETTI, M. & 4 others (1987*a*). Phosphorylation by protein kinase C of a 20 KDa cytoskeletal polypeptide enhances its susceptibility to digestion by calpain. *Proc. Natl Acad. Sci. USA.*, **84**, 398.

PONTREMOLI, S., MELLONI, E., MICHETTI, M. & 4 others (1987*b*). Phosphorylation and proteolytic modification of specific cytoskeletal proteins in human neutrophils stimulated by phorbol 12-myristate 13-acetate. *Proc. Natl. Acad. Sci. USA.*, **84**, 3604.

RACKOFF, W.R., RUBIN, R.A. & EARP, H.S. (1984). Phosphorylation of the hepatic EGF receptor with cAMP-dependent protein kinase. *Mol. Cell. Endocrin.*, **34**, 113.

RICHARDSON, J.M., MARLA, A.O. & WANG, J.Y.L. (1987). Reduction in protein tyrosine phosphorylation during differentiation of human leukemia cell line K-562. *Cancer Res.*, **47**, 4066.

ROSE, K., STETLER, D. & JACOB, S. (1981). Protein kinase activity of RNA polymerase I purified from a rat hepatoma: Probable function of Mr 42,000 and 24,600 polypeptides. *Proc. Natl Acad. Sci. USA.*, **78**, 2833.

SAHYOUN, N., WOLF, M., BESTERMAN, J. & 5 others (1986). Protein kinase C phosphorylates topoisomerase 11: Topoisomerase activation and its possible role in phorbol ester-induced differentiation of HL60 cells. *Proc. Natl. Acad. Sci. USA.*, **83**, 1603.

SATO, C., NISHIZAWA, K., NAKAJAMA, T. & 4 others (1986). Intranuclear appearance of the phosphorylated form of cytoskeleton-associated 350 kD protein in UI-ribonuclear regions after growth stimulation of fibroblasts. *Proc. Natl Acad. Sci. USA.*, **83**, 7287.

SCHLENDER, K.K., WEI, S.H. & VILLAR-PALASI, C. (1969). UDP-glucose alpha-4-glucosyltransferase 1 kinase activity of purified muscle protein kinase. Cyclic nucleotide specificity. *Biochim. Biophys. Acta.*, **191**, 272.

SEFTON, B., HUNTER, T., BALL, E. & SINGER, S. (1981). Vinculin: A cytoskeletal substrate of the transforming protein of Rous Sarcoma virus. *Cell*, **24**, 165.

SERFLING, E., JASIN, M. & SCHAFFNER, W. (1985). Enhancers and eukaryotic gene transcription. *Trends Genet.*, **1**, 224.

SHABB, J.B. & MILLER, M.R. (1986). Identification of a rat liver cAMP-dependent protein kinase, type 11, which binds DNA. *J. Cyclic. Nuc. Prot. Phosphor. Res.*, **11**, 253.

SHENOLIKAR, S., LICHTEIG, R., HARDIE, D.G., SODERLING, T.R., HANLEY, R.M. & KELLY, P.T. (1986). Calmodulin-dependent multifunctional protein kinase: Evidence for isoenzyme forms in mammalian tissues. *Eur. J. Biochem.*, **161**, 739.

SIBLEY, D.R. & LEFKOWITZ, R.J. (1985). Molecular mechanisms of receptor desensitization using the B-adrenergic receptor-coupled adenylate cyclase system as a model. *Nature*, **317**, 124.

SIBLEY, D.R., BENOVIC, J.L., CARON, M.G. & LEFKOWITZ, R.J. (1987). Regulation of transmembrane signaling by receptor phosphorylation. *Cell*, **48**, 913.

STRYER, L. & BOURNE, H.R. (1986). G-proteins: A family of signal transducers. *Ann. Rev. Cell. Biol.*, **2**, 391.

TAKAYAMA, S., WHITE, M.F., LAURIS, V. & KAHN, C.R. (1984). Phorbol esters modulate insulin receptor phosphorylation and insulin action in cultured hepatoma cells. *Proc. Natl Acad. Sci. USA.*, **81**, 7797.

TRIPP, H.L., PINON, R., MEISENHELDER, J. & HUNTER, T. (1986). Identification of phosphoproteins correlated with proliferation and cell cycle arrest in Saccharomyces cerevisae: Positive and negative regulation by cAMP-dependent protein kinase. *Proc. Natl Acad. Sci. USA.*, **83**, 5973.

UEDA, T. & PHAGENS, D.G. (1987). 3-phosphoglycerate-dependent protein phosphorylation. *Proc. Natl Acad. Sci. USA.*, **84**, 1229.

WEISINGER, G., KORN, A.P. & SACHS, L. (1985). Multimeric complexes of differentiation-inducing protein bound to DNA. *Eur. J. Cell. Biol.*, **37**, 196.

WEISINGER, G., KORN, A.P. & SACHS, L. (1986). Protein that induces cell differentiation causes nicks in double-stranded DNA. *Febs Lett.*, **200**, 107.

WILLIAMS, D.A., BECKER, P.L. & FAY, F.S. (1987). Regional changes in calcium underlying contraction of single smooth muscle cells. *Science*, **235**, 1644.

WOODGETT, J.R., GOULD, K.L. & HUNTER, T. (1986). Substrate specificity of protein kinase C: Use of synthetic peptides corresponding to physiological sites as probes for substrate recognition requirements. *Eur. J. Biochem.*, **161**, 177.

YANAGITA, Y., ABDEL-GHANY, M., RODEN, D., NELSON, N. & RACKER, E. (1987). Polypeptide-dependent protein kinase from bakers yeast. *Proc. Natl Acad. Sci. USA.*, **84**, 925.

YEN, A., FREEMAN, L. & FISHBAUGH, J. (1987). Hydroxyurea induces precommitment during retinoic induced HL-60 terminal myeloid differentiation: Possible involvement of gene amplification. *Leuk. Res.*, **11**, 63.

ZAJAC, J. (1984). Purification and some properties of protamine kinase from rabbit brain. *Acta. Biochim. Pol.*, **31**, 421.